# Added Value of Sensor-Based Behavioural Monitoring in an Infectious Disease Study with Sheep Infected with *Toxoplasma gondii*

**DOI:** 10.3390/ani14131908

**Published:** 2024-06-27

**Authors:** Harmen P. Doekes, Ronald Petie, Rineke de Jong, Ines Adriaens, Henk J. Wisselink, Norbert Stockhofe-Zurwieden

**Affiliations:** 1 Animal Breeding and Genomics, Department of Animal Sciences, Wageningen University & Research, P.O. Box 338, 6700 AH Wageningen, The Netherlands; 2 Wageningen Bioveterinary Research, Wageningen University & Research, 8221 RA Lelystad, The Netherlands; 3 Research Group BioVism, Department of Data Analysis and Mathematical Modelling, Ghent University, Coupure Links 653, 9000 Ghent, Belgium; 4 Livestock Technology Group, Department of Biosystems, Division of Animal and Human Health Engineering, Kleinhoefstraat 4, 2440 Geel, Belgium

**Keywords:** sheep, behaviour, welfare, scientific endpoints, humane endpoints, computer vision

## Abstract

**Simple Summary:**

The aim of this study was to investigate the added value of two sensor technologies (accelerometers and video) to monitor the activity and drinking behaviour of three sheep infected with the parasite *Toxoplasma gondii*. We found that the sensor technologies offered additional insights as compared to routine observations. First, they provided 24/7 behavioural records, instead of the bidaily snapshots from human observations. Second, they provided individual data. For example, they showed that each sheep spent 50% less time at the drinker after infection, while the traditional water meter gave only results at the group level. Third, they provided a quantitative measure. For example, they showed that each sheep was from 20% to 40% less active after the infection, while the animal caretakers only scored whether animals were lethargic or not. Last, they provided objective behavioural patterns. For example, they showed that daytime activity was reduced from day 4 to day 10 after infection, while the caretakers only recorded that the sheep were lethargic from day 5 to day 7. While we recognize the challenges and pitfalls of sensor technologies, we recommend wider implementation in animal disease trials to refine experiments and guarantee the quality of their results.

**Abstract:**

Sensor technologies are increasingly used to monitor laboratory animal behaviour. The aim of this study was to investigate the added value of using accelerometers and video to monitor the activity and drinking behaviour of three rams from 5 days before to 22 days after inoculation with *Toxoplasma gondii*. We computed the activity from accelerometer data as the vectorial dynamic body acceleration (VDBA). In addition, we assessed individual drinking behaviour from video, using frame differencing above the drinker to identify drinking bouts, and Aruco markers for individual identification. Four days after inoculation, rams developed fever and activity decreased. The daytime VDBA from days 4 to 10 was 60–80% of that before inoculation. Animal caretakers scored rams as lethargic on days 5 and 6 and, for one ram, also on the morning of day 7. Video analysis showed that each ram decreased its number of visits to the drinker, as well as its time spent at the drinker, by up to 50%. The fever and corresponding sickness behaviours lasted until day 10. Overall, while we recognize the limited conclusiveness due to the small number of animals, the sensor technologies provided continuous, individual, detailed, and objective data and offered additional insights as compared to routine observations. We recommend the wider implementation of such technologies in animal disease trials to refine experiments and guarantee the quality of experimental results.

## 1. Introduction

Monitoring the behaviour of laboratory animals is an important strategy to improve the quality of experimental results and refine animal experiments [1,2,3]. First, it can help to better understand the effect of an experimental treatment. In infectious disease studies, for example, behavioural patterns provide additional information on the onset of disease, disease development, disease transmission, and the effectiveness of treatments [4,5,6]. Second, it can help to better detect scientific endpoints. When (unexpected) behavioural anomalies occur, either caused by the treatment or by confounding factors, a scientific endpoint may be desired to safeguard the validity and reliability of experimental results. Last, given that behaviour is an important indicator of an animal’s wellbeing, continuous behavioural monitoring allows for timely intervention when an animal’s welfare is impaired. When the impairment is severe, a humane endpoint may be applied [3,7].

Traditionally, routine monitoring procedures rely on short (bi-)daily visual observations of the animals by researchers and animal caretakers. Visual observations are a valuable source of information, but also have limitations. First, only snapshots are taken, typically during the daytime. Consequently, important (changes in) behaviour may be missed, especially for species that are crepuscular or nocturnal. Moreover, when anomalies arise, their exact onset is unknown. Second, inter- and intra-observer biases decrease the reliability of human observations [8,9]. Multiple observers may interpret the same behaviour differently and a single observer may interpret the same behaviour differently over time, e.g., due to habituation based on prior observations. Last, the presence of an observer may influence the animal’s behaviour [10,11], reducing the validity of the measurements. These limitations could be overcome with continuous and automated monitoring procedures. 

The continuous and automated monitoring of behaviour has become increasingly possible due to the rapid developments in sensor technologies [12,13]. A myriad of sensor technologies exist, and here we focus on those that can be used to monitor individual behaviour, specifically activity, in somewhat larger (i.e., non-rodent) laboratory animals in group housing. For this application, the commonly used technologies can be divided into two categories: (1) body-worn sensors and (2) computer vision (CV). 

Body-worn sensors, such as accelerometers, ultra-wideband (UWB) sensors, and radio-frequency identification (RFID) tags, store data on the device itself and/or send signals to a receiver for storage elsewhere. Each sensor has its pros and cons in terms of user-friendliness, costs, size, weight, battery life, water- and dust-proofness, and interference with metal, water, and pen mates, among others (e.g., [12]). Triaxial accelerometers measure acceleration in three orthogonal directions (x, y, and z) and have been applied to assess overall activity and specific behaviours in many species [13,14,15]. They are widely applied due to their lightness and relatively high user-friendliness, with no need to install additional hardware. The latter is an important advantage for in vivo infection experiments that last a few weeks and/or are performed in high containment conditions, where all hardware needs to be removed, disinfected, and re-installed after each experiment. 

Computer vision (CV) refers to the use of cameras to record animal behaviour. There is a wide range of CV techniques available, of which neural networks are increasingly developed to detect individual animals in a group over time. For laboratory rodents, home-cage monitoring systems have been developed [16,17]. For other species in less standardized environments, however, (re-)identification and long-term tracking remains very challenging (e.g., [18]). To facilitate (re-)identification, one may use markers on the animals, such as coloured ear tags or Aruco markers [19,20], or RFID [21]. However, other challenges still remain, such as occlusion, and, in the case of larger pens, the tracking of animals across large surface areas and multiple cameras [22]. Thus, for the time being, body-worn sensors may provide more guaranteed continuous time series for animal monitoring.

Despite their potential added value, sensor technologies are not yet widely adopted in animal experiments as part of routine monitoring procedures. Reasons for the limited implementation may be manifold, including limited expertise with the available technologies and the required data analysis costs, experimental concerns (impact on validity of experimental results), and ethical concerns (e.g., impact on the animal). Likely, as with any innovation, sensor technologies must first prove their added value before they will be widely adopted in animal experiments. 

In this study, we used sensor technologies to monitor the behaviour of rams in an infectious disease study with *Toxoplasma gondii*, a parasite that causes the zoonotic disease toxoplasmosis. The mean prevalence of toxoplasmosis in worldwide livestock populations has been estimated at 28.3% in recent decades [23]. A large part (if not the largest part) of transmission to humans occurs through the consumption of meat products of infected animals and, in the Netherlands, >80% of meat-borne infections have been predicted to be due to the consumption of undercooked (particularly beef, pig, or sheep) meat containing tissue cysts [24]. 

The aim of this study was to investigate the added value of two sensor technologies (accelerometers and video) in monitoring the activity and drinking behaviour of three rams from 5 days before to 22 days after inoculation with *T. gondii*. We compared the insights obtained from these sensor technologies to those obtained through the routine monitoring procedures. We also reflected on the effect of the infection on sheep behaviour.

## 2. Materials and Methods

### 2.1. Animals and Experimental Treatment 

**Figure 1:**
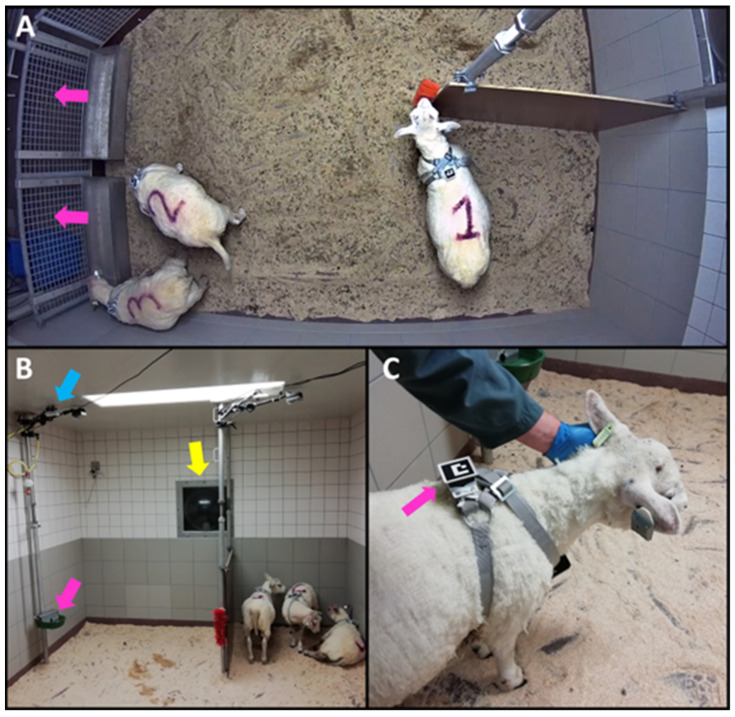
Overview of the pen and sensors. (**A**) Top view of the pen with corridor for animal caretakers indicated by pink arrows. (**B**) Drinker, camera above the drinker, and bubble window for observations by animal caretakers indicated by pink, blue, and yellow arrows, respectively. (**C**) Accelerometer and Aruco marker indicated by pink arrow.

Three Texelaar cross rams of seven months old were used. These rams were purchased from a conventional sheep herd in the Netherlands with no recent *T. gondii* abortion history, no use of a vaccine against *T. gondii*, and no (young) cats present. The rams arrived at the research facility seven days before the inoculation (‘day −7’) and tested seronegative for *T. gondii* (for details on testing, see [24]). The rams were housed in a pen of 10.9 m^2^ with a wooden semi-partition, sawdust as bedding, a drinker, feeding bins, and a scratching brush (Figure 1A,B). Throughout the experiment, the temperature in the pen varied from 18.9 °C to 20.2 °C and relative humidity varied from 54% to 70%. Lights were on daily from 6 am to 6 pm.

After one week of acclimatization, rams were orally inoculated (on ‘day 0’) at 11:00 with 5 mL of 2 × 10^3^ oocysts/mL of the Savannah strain of *T. gondii* using a curved needle with a blunt bulb. Details on the approach, including the preparation of the oocysts, can be found in Opsteegh et al. [24]. The aim was to produce infected meat for subsequent optimization of a previously developed in vitro model for the detection of viable *T. gondii* in raw or insufficiently heated meat. At 10 weeks (one ram) and 18 weeks (two rams) after inoculation, the sheep were euthanized using an intravenous pentobarbiturate overdose (in the vena jugularis) followed by exsanguination (according to EU Directive 2010/63 ANNEX IV) and necropsy/dissection to obtain muscle tissue specimens. To assess whether rams were chronically infected, a pooled sample of six tissue specimens derived from heart, diaphragm, and muscles were digested with a pepsin-HCl solution followed by a quantitative polymerase chain reaction on the digest (similar to [24]).

**Figure 2:**
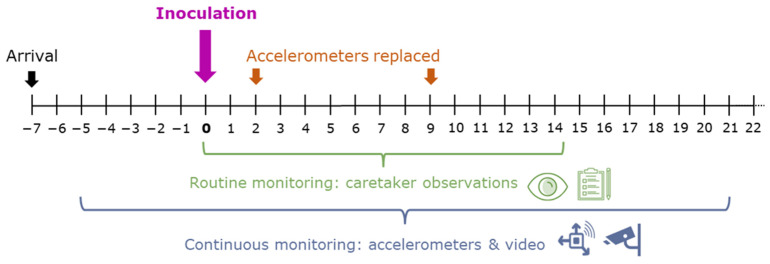
Timeline of the experiment, with 27 days of data recording around inoculation (‘day 0’, purple). Routine monitoring (green) included (bi-)daily scoring of appetite, activity, respiration rate, and body temperature by caretakers, as well as water consumption by the group (from day −4 to 22; not shown in figure). Continuous monitoring (blue) included monitoring of activity and drinking behaviour with accelerometers and video, with accelerometers being replaced twice (orange).

Clinical symptoms were expected to occur only during the first two weeks after inoculation [25,26]. To reduce clinical symptoms, meloxicam (1 mg/50 kg; Novem 20, Boehringer Ingelheim Vetmedica GmbH, Ingelheim am Rhein, Germany) was administered intramuscularly (in gluteal muscles) when a ram developed fever. Fever was defined as rectal temperature above 41 °C observed twice at an observation interval of 24 h, with normal temperatures for sheep ranging from 38.5 to 40 °C. Routine monitoring measurements were taken from the day of inoculation to two weeks after inoculation (day 0 to day 14), and additional sensor measurements were taken from approximately a week before to three weeks after inoculation (from day −5 to day 22). The monitoring timeline is summarized in Figure 2 and further explained below. 

### 2.2. Routine Monitoring: (Bi-)Daily Observations by Animal Caretakers

As part of the routine monitoring procedures, water consumption and appetite were scored daily, with intervals varying from 21 to 27 h. Water consumption at group level was registered from day −4 to day 22 with a water meter in the drinker and was rounded to entire litres. Rams were fed a combination of high energy pellets, grass pellets, and hay. Animal caretakers recorded leftovers from day 0 until day 14 and, when providing feed, registered whether individual rams showed a reduced appetite.

From day 0 until day 14, animal caretakers also performed bidaily measurements, in the morning (between 7:00 and 10:00) and afternoon (between 13:00 and 16:00). Body temperature was measured using a rectal thermometer, respiration rate (breaths per minute) was estimated by observing the movement of flanks, and the activity of rams was determined by visual observation and scored as ‘normally active’ or ‘lethargic’. 

### 2.3. Activity Based on Accelerometers

To assess the added value of continuous activity monitoring, we collected accelerometer data from day −5 (starting at 5 p.m.) until day 22 (ending at 10 a.m.). We equipped each ram with a MOX1 accelerometer (Maastricht Instruments BV, Maastricht, The Netherlands). We placed the accelerometer in a 3D-printed box on the back of the ram, which we attached to a mating harness from which the marking crayon slot was removed (Figure 1C). The accelerometers were 35 × 35 × 10 mm, weighed 11 g, had a dynamic range of ±8 g, and measured x-, y-, and z-acceleration at 25 Hz with a 15-bit resolution. Given their battery life of 1–2 weeks, we recharged and replaced the devices on days 2 and 9 (Figure 2). When replacing a device, we reattached it in the same orientation, and to the same animal, as before the replacement. 

We merged the data of consecutive recording sessions (i.e., from day −5 to day 2, day 2 to day 9, and day 9 to day 22) and discarded the hours at which accelerometers were replaced. We then computed the vectorial dynamic body acceleration (VDBA) as a measure of overall activity. The VDBA is preferred over the overall dynamic body acceleration (ODBA) introduced by Wilson et al. [27] when a constant device orientation cannot be guaranteed over time and/or between animals [28,29]. The VDBA was calculated as:

(1) $${\mathrm{VDBA}}_{i}=\sqrt{{\left(x_{i}-{\bar{x}}_{i}\right)}^{2}+{\left(y_{i}-{\bar{y}}_{i}\right)}^{2}+{\left(z_{i}-{\bar{z}}_{i}\right)}^{2}}$$

where $x_{i}$, $y_{i}$, and $z_{i}$ were the raw accelerations at time *i*, and (${\bar{x}}_{i}$), (${\bar{y}}_{i}$), and (${\bar{z}}_{i}$) were the rolling means of the acceleration signals for the three axes (in *g*). We used an interval of 2 s (i.e., 50 measurements), similar to previous work in sheep [30]. To facilitate the interpretation of activity patterns across the experiment, we calculated the mean VDBA per hour and smoothed the pattern with a centred rolling mean of 8 h.

### 2.4. Time Spent at the Drinker Based on Video

To continuously monitor drinking behaviour, we installed an AXIS M1065-L network camera (Axis Communications AB, Lund, Sweden) above the drinker, together with an infrared illuminator for night vision (Figure 1B). The camera recorded consecutive sessions of 24 h using Mediarecorder software version 6.0 (Noldus Information Technology, Wageningen, The Netherlands), starting daily around 4 p.m. with a resolution of 1920 × 1072 pixels and a frame rate of 25 fps. Unfortunately, recordings that started on day −4 and day 15, and parts of the recordings that started on day 3 and day 16, were corrupted and could not be used.

For each video, we estimated the time each ram spent at the drinker by combining two computer vision approaches: (1) frame differencing to detect motion above the drinker (e.g., [31]), and (2) Aruco markers to identify which individual was present at the drinker (e.g., [19]). To reduce computation time, the videos were down sampled to ~2 fps. 

**Figure 3:**
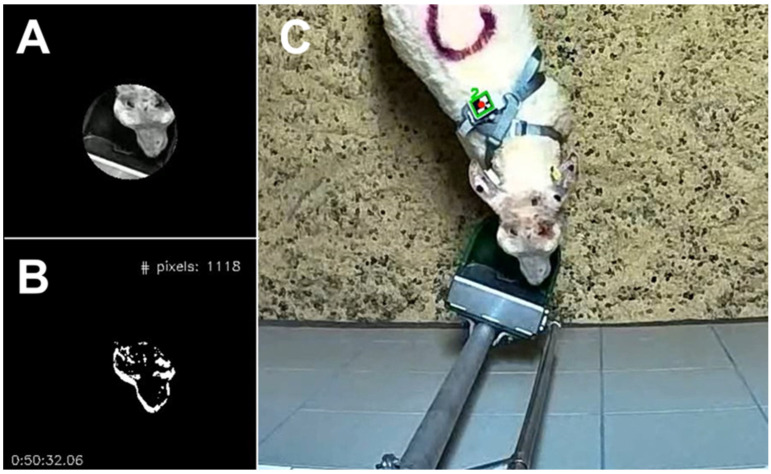
Analysis of time spent at drinker with frame differencing and Aruco markers. (**A**) Mask applied on the drinker. (**B**) Pixels that significantly changed in intensity compared to previous frame shown in white. (**C**) Detection of the Aruco marker in the vicinity of the drinker.

To detect motion above the drinker, we first extracted the drinker as a circle with a radius of 56 pixels and an area of 9852 pixels (Figure 3A,B). We changed the location of the mask thrice throughout the period, to correct for slight position changes caused by rams bumping into the drinker. We converted each frame to grayscale, resulting in intensity values ranging from 0 through to 255, and then computed the number of pixels above the drinker that ‘significantly’ changed in intensity value for any two consecutive frames, using a threshold of >70 in intensity value. As preliminary analysis, we used one 24 h video and extracted all the frames for which more than 100 of the 9825 pixels (i.e., >1%) changed significantly. This was the case for 2224 frames (~0.1% of all frames). We then manually checked the video to determine whether these frames represented an animal drinking. While doing so, we identified 34 drinking bouts, which varied in duration from approximately 5 to 71 s (Table S1). While manually annotating these drinking bouts, we allowed bouts to consist of one sip or multiple sips with some time in between (up to 10 s) during which the ram would not be actively consuming water (as far as this was possible to tell). The 34 identified bouts showed a common pattern, with typically many pixels changing at the start and end of a bout when the animal would approach or leave the drinker, and fewer but nonzero pixels changing in the middle of the bout when the animal remained approximately still with its head above the drinker. Based on these preliminary findings, we defined a “drinking bout” to start with substantial movement above the drinker (defined as a rolling average of 5 frames of ≥800 pixels changed) and to end when movement was absent for more than 5 s (i.e., a moving average of 10 frames that dropped below 15). When applying these criteria to the validation video, we detected all 34 manually annotated drinking bouts, thus obtaining a ‘sensitivity’ of 100%. For two cases, a single bout was detected consisting of two manually annotated bouts. The lengths of the detected bouts correlated reasonably well (*r* = 0.75) with the lengths of the manually annotated bouts, even though the former tended to be somewhat longer (Table S1). There were 18 false positive bouts and the vast majority (13 out of 18) of these false positives were caused by rams moving their heads above the drinker without taking a sip. Since these false positives were impossible to distinguish from true positives, we considered the set criteria to be sensible and, using these criteria, we automatically detected 1651 “drinking bouts” in all videos. We discarded bouts that occurred during the 10 min in which the lights turned on in the morning (from 5:55 to 6:05) or off in the evening (from 17:55 to 18:05), as they were deemed false positives caused by light intensity changes. Similarly, we discarded bouts from the 40 min period of inoculation (from 10:50 to 11:30 on day 0), because there was a lot of human activity in the pen during this period, including above the drinker. For the final analysis, 1605 bouts remained. 

To assign drinking bouts to individual rams, we used Aruco markers that were attached on top of the 3D-printed case in which the accelerometer was placed (Figure 1C). The Aruco markers were from the “DICT_4 × 4_50” dictionary of OpenCV in Python and were printed on waterproof paper. Based on the 34 manually identified drinking bouts, we selected an area of 600 by 600 pixels around the drinker that always contained the Aruco marker of the ram that was drinking (Figure 3C). For each bout, we detected Aruco markers using the “detect_Aruco” function in OpenCV [32] and assigned the bout to individual rams. When multiple rams were detected in the vicinity of the drinker (*n* = 259 bouts, ~16% of all bouts), the total duration of the bout was partitioned across the rams proportionally to their number of detections to minimize the error for the involved rams. For example, when, for a bout of 40 frames, ram 1 was detected in 25 frames, and ram 2 was also detected in 25 frames, both rams were assigned a drinking bout of 20 frames. Thus, we assumed that rams would not drink at the same time, which we deemed likely for most bouts considering the size of the drinker, even though in one out of the 34 annotated drinking bouts two rams were drinking simultaneously (Table S1). No Aruco marker was detected for 75 bouts (~5% of all bouts). Most of these bouts occurred in the first week (*n* = 59) and, after manually checking part of these bouts, we assigned them all to ram 3, because the Aruco marker of ram 3 was often not visible in the first recording week due to a loose strap of the mating harness (which was removed on day 2). The remaining 16 bouts with an unknown ram were discarded for the further analysis. 

Finally, we calculated the number of visits to the drinker, as well as the time spent above the drinker per ram per hour, and visualized results as a rolling mean of 8 h. 

### 2.5. Statistical Analysis

To assess whether the behaviour of the rams (VDBA, time spent at drinker, and number of drinking bouts) significantly changed after the inoculation with *T. gondii*, we used two approaches. First, at the individual level, we compared each ram’s observed behaviour per hour after the infection with its expected behaviour. The expected behaviour was based on the ram’s mean behaviour for the corresponding hour in the days before the infection, as well as its minimum and maximum. Second, at the group level, we performed two-sided paired *t*-tests to compare whether—for the group of three rams—the behaviour for each day after the infection significantly differed from the mean behaviour in the days before infection. An alpha of 0.05 was used as a threshold for statistical significance. 

## 3. Results

### 3.1. Routine Monitoring Results

**Figure 4:**
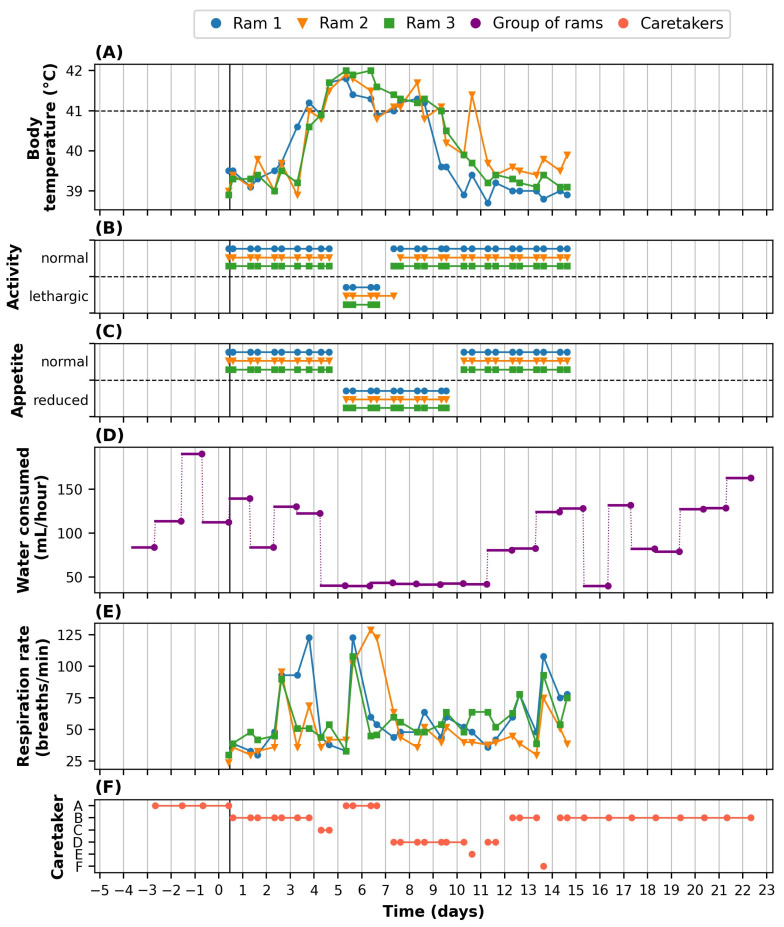
Routine monitoring results for three rams from 5 days before to 22 days after inoculation with *T. gondii*. The vertical black line on day 0 indicates the exact moment of inoculation. (**A**) Rectal temperature of each ram. (**B**) Activity of each ram scored as normal or lethargic. (**C**) Appetite of each ram scored as normal or reduced. (**D**) Water consumed by the group of rams, recorded through a water meter once a day but expressed as average per hour. (**E**) Respiration rate of each ram scored by observing movement of flanks. (**F**) Animal caretaker schedule.

All rams developed fevers from three to four days after inoculation, reaching a peak body temperature of approximately 42 °C on day 5 (Figure 4A). To reduce their fevers, the rams were treated with meloxicam from day 4 to day 7. The fevers lasted until day 8 (for ram 1) and day 9 (rams 2 and 3). 

All rams were scored to be lethargic on days 5 and 6, and ram 2 was also scored to be lethargic in the morning of day 7 (Figure 4B). Based on observations of feeding behaviour and leftovers, a reduced appetite was recorded for all rams from day 5 to day 9 (Figure 4C). Daily readings from the water meter showed that, prior to inoculation, the three rams consumed on average 125 mL/h as a group (Figure 4D). A threefold reduction occurred on day 4, after which the rams consumed approximately 42 mL/h as a group. This lasted until day 12, after which the rams were back to a similar level as before the inoculation, with on average 106 mL/h from day 12 to day 22. The respiration rate in the first days after inoculation (i.e., before the fever developed) was approximately from 30 to 40 breaths per hour for each ram (Figure 4E). Throughout the two weeks of monitoring, the respiration rate was scored above 90 breaths/min (i.e., a threefold increase) a few times, but never for more than three subsequent measurements. 

Throughout the monitoring period, six different animal caretakers were involved in the routine monitoring, though 85% of all measurements were performed by three of the caretakers (Figure 4F). The caretakers typically rotated every 2–3 days, except for the measurements taken from day 14 to day 22, which were performed by a single caretaker.

### 3.2. Activity Based on Accelerometers

**Figure 5:**
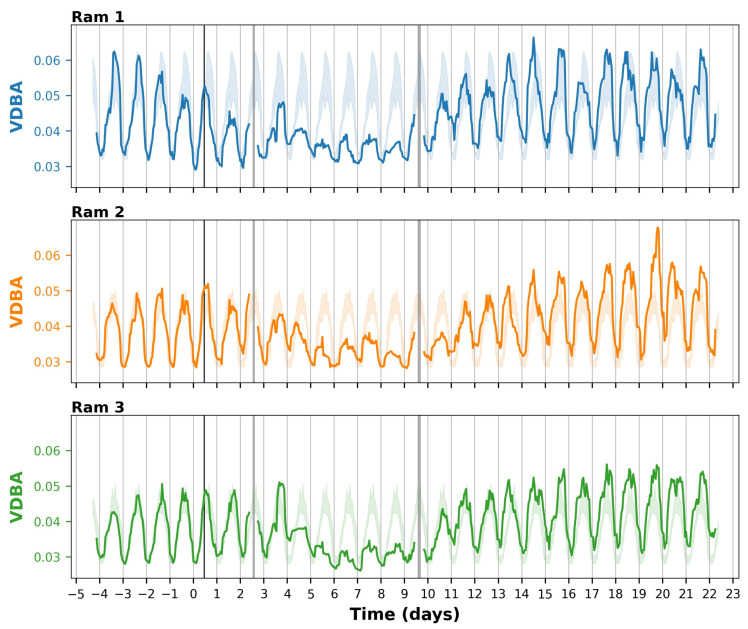
Vectorial dynamic body acceleration (VDBA) of three rams from 5 days before to 22 days after inoculation with *T. gondii*. The VDBA is presented as a centred rolling average of eight hours. The vertical black line on day 0 indicates the exact moment of inoculation. The shaded coloured areas in the background indicate the range (from minimum to maximum) of VDBA levels of the corresponding hours in the days before infection. Vertical grey bars indicate the moments when accelerometers were replaced.

Prior to inoculation, there was a clear circadian rhythm in the accelerometry-based activity for all three rams (Figure 5). The VDBA across rams and days was from 0.3 to 0.35 during the most inactive hours in the night (typically from 23:00 to 03:00) and from 0.43 to 0.62 during the most active hours during the day (typically from 11:00 to 20:00). The VDBA during the daytime was 1.5–2 times higher than during the night. 

On day 4 after the inoculation, the activity pattern changed, and the circadian rhythm became less pronounced (Figure 5). For each ram, the daytime VDBA decreased to 60–80% of what it used to be before inoculation, falling below the ‘normal’ range (minimum–maximum) of observed values before the inoculation (Figure 5 and Figure S1). The night-time VDBA remained approximately the same. This pattern continued until day 9 (for ram 1) and day 10 (for rams 2 and 3), which was in line with the recovery from the fever (Figure 4A). At the group level, the mean VDBA per day was also significantly reduced (*p* < 0.05) from day 5 to day 8 and tended to be significantly reduced (*p* < 0.10) on day 4 and 10, with day 9 not being tested due to incomplete data (Table 1). In the long term, in the period from day 11 to day 23, the activity levels were systematically higher than before the inoculation, especially during the daytime (Figure 5, Table 1). Specifically, from day 11 to day 22, the VDBA during the daytime peak was 110–160% of that before inoculation (Figure S1).

### 3.3. Time Spent at the Drinker Based on Video

**Figure 6:**
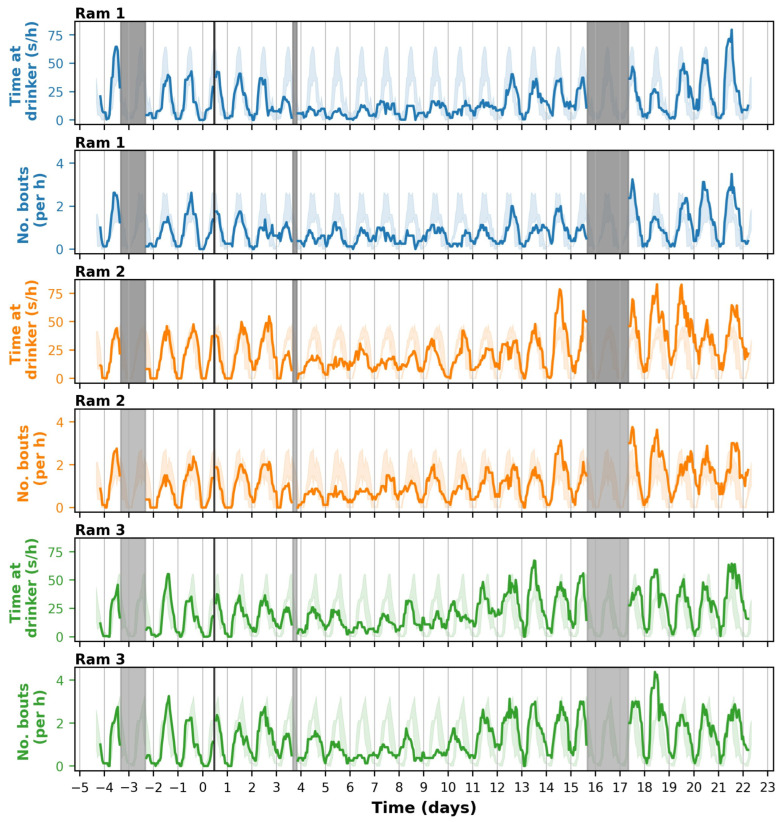
Drinking behaviour of three rams from 5 days before to 22 days after inoculation with *T. gondii*. Both the time spent at the drinker (in seconds per hour) and the number of drinking bouts (per hour) are presented as a centred rolling average of eight hours. The vertical black line on day 0 indicates the exact moment of inoculation. The shaded coloured areas in the background indicate the range (minimum–maximum) of levels of the corresponding hours in the days before infection. Vertical grey bars indicate corrupt video segments with no drinking behaviour detected.

The number of visits to the drinker and the time spent at the drinker were rather similar for the different rams (Figure 6). The number of bouts and the time spent at the drinker showed circadian rhythms, with each ram visiting the drinker primarily during the daytime, i.e., between 6:00 and 20:00. Prior to inoculation and during daytime peaks, each ram visited the drinker approximately twice every hour and spent approximately 40 s at the drinker. On day 3 after inoculation, this decreased to one visit or less and to 20 s per hour (Figure 6 and Figure S2). Thus, the number of visits and the time spent at the drinker approximately halved, falling below the ‘normal’ range (minimum–maximum) of the observed values before the inoculation. At the group level, the number of visits and the time spent at the drinker were also significantly reduced (*p* < 0.05), by up to 50%, in most of the days between day 4 and 10. The reduction in time spent at the drinker lasted until day 10–12 depending on the ram. Although each ram visited the drinker less frequently from day 3 to day 12, they did not completely stop going to the drinker. 

From day 11 onwards, the number of visits to and the time spent at the drinker increased as compared to the mean before inoculation (Table 1, Figure 6 and Figure S2). Towards the end of the monitoring period, from days 18 to 21, the number of visits and the time spent at the drinker were significantly higher than before infection, especially during the daytime activity peaks (Table 1, Figure S2). During the daytime peaks, each ram performed approximately one additional drinker visit per hour and spent approximately 30 more seconds at the drinker than in the corresponding hours before the infection (Figure S2). 

## 4. Discussion

In this study, we investigated the added value of continuous and automated behavioural monitoring in infectious disease studies with farm animals. We used a case study in which we implemented two sensor technologies, accelerometers and video, to monitor activity and drinking behaviour in rams infected with the parasite *T. gondii*. We studied the effects of the *T. gondii* infection on the behaviour of the rams and compared the results of routine monitoring procedures (i.e., bi-daily short observations by animal caretakers) with those of the continuous monitoring (i.e., sensor technologies) to assess the added value of the latter, as well as the challenges. 

### 4.1. Effects of T. gondii Infection on the Behaviour of Sheep

The physiological clinical symptoms we observed, such as the fever that the rams developed and some signs of respiratory distress (Figure 4A,E), have been previously reported in various studies with sheep experimentally infected with *T. gondii* oocysts [25,26]. Behavioural results, however, have rarely been reported in the literature. 

We observed various changes in the rams’ behavioural patterns that coincided with the period of fever (Figure 4A). The substantial decrease in activity, with the daytime VDBA decreasing to 60–80% of what it used to be (Figure 5), is a typical “sickness behaviour” associated with fever. Fever is part of a systemic response that helps to combat infection but is metabolically costly [33]. Reducing locomotor activity helps to save metabolic energy [33,34,35]. Similarly, the decreased appetite from day 5 to day 10 (Figure 4C) is a well-known sickness behaviour that saves energy which is otherwise allocated to feeding and digestion [33,34]. We also observed a strong reduction in drinking behaviour, with the amount of water consumed by the group (Figure 4D) and the individual time spent at the drinker (Figure 6, Table 1) both decreasing by approximately 50% on days 4–11. Given that the number of drinking bouts showed a similar trend as the time spent at the drinker (Figure 6), the decrease in time spent at the drinker was primarily caused by rams visiting the drinker less frequently, rather than by shorter drinking bouts. A reduced motivation for drinking after infection has been previously observed and is likely a result of shifting priorities [36]. A classic example is the experiment of Miller [37], who found that rats treated with endotoxins pressed a lever for rewards (including food and water) less frequently, whereas they increasingly pressed a lever to get periods of rest when they were forced to run in a wheel. Verheijden et al. [38] observed that six pigs, after being challenged with *Actinobacillus pleuropneumoniae* toxins, consumed 25–50% of the amount of water that they consumed before infection. The reduced water intake lasted until three days after the challenge. Similarly, Ahmed et al. [39], using 2 h recordings, found that pigs infected with Salmonella strains had significantly lower drinking frequencies (typically 50% or even less) than a control group in the morning, but not in the evening. The observed reduction in drinking frequency following infection, as observed in these infectious disease studies, is likely linked to the reduction in feeding, given that many animals tend to drink after feeding [40,41]. Still, the strong effects of infectious diseases on drinking behaviour are interesting for further research and emphasize the relevance of monitoring drinking behaviour in laboratory and other settings. 

After the initial decrease in activity associated with the febrile response, the rams did not return to the same activity levels as before inoculation, but the VDBA for all rams increased to 110–160% of what it used to be (Table 1, Figure 5 and Figure S1). This might be a long-term effect of the infection with *T. gondii*. The qPCR at the end of the experiment confirmed that all three rams were chronically infected, with *T. gondii* being detected in the pepsin-HCL-digested pooled sample of heart and muscle tissue specimens of each ram. It is known that cysts can be chronically traced in muscle, heart, and brain tissue, with a predilection for the brain [25]. It seems plausible that this may lead to behavioural changes, even though the biological mechanism remains largely unknown [42,43,44]. One possibility is that the local infection of heart and muscle tissue (myositis) may cause subclinical pain and restlessness in the animals. Alternatively, immunological and/or neuromodulatory changes may play a role. In mice and rats, it has been shown that *T. gondii* alters host hormone levels, alters neurotransmission within the host brain, and may affect neuroinflammation [43,44]. Moreover, in these rodents, infected hosts show an increased activity, decreased neophobic behaviour, and decreased vigilance for predators [42]. It is hypothesized that *T. gondii* has evolved such that it induces these behavioural changes in the intermediate hosts to stimulate transmission to cats (the definitive host in which sexual reproduction can occur), thereby completing its life cycle [42]. In humans, an end-stage intermediate host, toxoplasmosis has also been associated with a range of behavioural alterations and conditions, but whether these links are causal or simply correlational is a subject of debate [45]. In sheep, another end-stage intermediate host, there are also indications that infection with *T. gondii* may induce behavioural alterations. Shamsi et al. [46] reported that sheep seropositive for *T. gondii* were slower to solve a spatial maze than seronegative sheep. However, they did not observe a difference in fear response between seropositive and seronegative sheep [46]. Our results suggest that infection with *T. gondii* may lead to a long-term increase in activity in sheep, similar to what is reported for mice and rats, but we should note that our study design (with few animals, no control group, a single environment, and only 3 weeks post-infection) does not allow us to draw firm conclusions. Further studies, with larger sample sizes and neuroendocrine measurements, are needed to obtain more conclusive results on the effect of infection with *T. gondii* on the short-term and long-term behaviour of sheep and other species, and to better understand its physiological mechanism. 

### 4.2. Added Value of Sensor Technologies

The implemented sensor technologies provided additional insights into the short-term and long-term effects of the infection on the animals’ behaviour, as compared to the routine monitoring results. First, the sensor technologies provided continuous behavioural patterns, in addition to the (bi-)daily snapshots. With the sensor technologies, we showed that activity during the daytime dropped as part of the acute infection response, whereas the night-time activity levels remained rather constant (Figure 5), the latter of which was not assessable with the routine monitoring procedure. Similarly, we showed that the rams visited the drinker mostly during the daytime (Figure 6). To assess general activity patterns, we used a rolling average of 8 h, but one could even further zoom in to investigate peaks across the day, for example to assess whether animals primarily drank during or directly after feeding, as may be expected based on the literature [40,41]. 

Second, the sensor technologies provided individual behavioural patterns. With our CV approach, we showed that each ram continued to visit the drinker after inoculation (Figure 6), albeit at a lower frequency than before inoculation, whereas the routine monitoring procedure (i.e., the water meter in the drinker) only measured consumption at the group level (Figure 4D). Thereby, we showed that one of the pre-defined humane endpoints of “not being able to independently move to the drinker” was not met for any of the rams. This was impossible to conclude from the routinely collected group level data. In general, we believe that individual (behavioural) time series are essential for monitoring, considering that baselines may differ substantially, for example between species, breeds/strains, sexes, ages, housing conditions, ways of attaching the sensor, and individuals. 

Third, the sensor technologies provided more objective behavioural patterns. The accelerometer data indicated a reduced daytime activity from day 4 to day 9 for ram 1, and from day 4 to day 10 for rams 2 and 3 (Figure 5). Interestingly, the animal caretakers only scored the three rams to be lethargic on days 5 and 6, and ram 2 also on the morning of day 7 (Figure 4B). Given that the observations on days 5 and 6 were performed by caretaker A, those on day 4 by caretaker C, and those on days 7–10 by caretaker D (Figure 4F), we expect that inter-observer variability may have played a role. Alternatively, the presence of a caretaker may have increased the activity of the animals, although previous studies in (mostly wild) animal species tend to report negligible observer effects or observer effects in the opposite direction [10,11,47]. As another possibility, the snapshots from days 7 to 10 may have coincided with “active moments” of the animals by chance. Since “being lethargic” is typically also part of scientific and humane endpoints, it is important that the monitoring of activity levels is objective to prevent incorrect decision-making. 

Fourth, the sensor technologies provided more detailed insights. For example, the routine monitoring involved a binary score of activity, namely ‘normal’ or ‘reduced’ (lethargic), whereas the accelerometers provided continuous measurements, e.g., the VDBA reducing to 60% of what it used to be. Note that we focused on approaches that can be generally applied to a variety of species and experiments, but that sensor technologies also allow quantifying more specific behaviours when (species-specific) models are trained and applied (e.g., [48,49]), allowing for even more detailed insights. 

Last, the sensor technologies could be combined with direct and automated data processing, which could then be used to generate warning signals when anomalies arise (e.g., [49]). Thanks to automatization, the workload is low once the system is in place, and it is relatively easy to extend the monitoring period. In our study, routine monitoring was performed for the two weeks after infection since most clinical symptoms were expected in this period (Figure 2), whereas the sensor technologies were also used the week before the infection and the third week after infection. As a result, we detected the increase in activity after day 10, which we would otherwise have missed. Thus, through automatization, we can more easily extend the monitoring period and capture, for example, acclimatization and long-term treatment effects.

### 4.3. Challenges of Sensor Technologies 

Obviously, sensor technologies also have their challenges. A first challenge for wider application is the diversity of (experimental) systems, which includes variation in the species, breeds, and ages of the animals. The species and age, for example, determine if, and how, a sensor (or marker) can be attached to the animal. In addition, they determine which specific behaviours are expressed and—in combination with the environment—in which manner. While general activity (e.g., through VDBA) may be measurable in a generalized way across species, the detection and monitoring of specific behaviours (e.g., feeding or drinking) will require species-specific (or even study-specific) model development and validation. 

Second, the use of sensor technologies gives rise to (new) ethical questions. The most common concern is the impact of body-worn sensors on an animal’s physical status and its ability to perform natural behaviour [50,51]. In our study, based on visual observations, the rams seemed to be hardly affected by the mating harness and the ear-based accelerometer. There were no signs of physical harm nor clear behavioural alterations, aside from a short habituation period of less than 5 min. Given the weight of the accelerometers (11 g) and the weight of the rams (approximately 30 kg), we stayed well below the common rule of thumb of the sensor weighing less than of 5% of the animal’s body mass [52]. Nevertheless, we cannot guarantee that the sensors did not affect the animal’s affective states. Moreover, for smaller animals, or in cases when multiple sensors are used, it will be more challenging to limit the impact on the animal. Aside from the impact of sensors on an animal’s physical health and behaviour, other ethical concerns include, among others, (1) a loss of personal connection between human caretakers and animals due to overreliance on the technology, which could impact the animal’s wellbeing; (2) a further objectification of animals as mere data points; and (3) shallow use of the data for better results, such as higher production efficiency in commercial livestock, or better experimental results in animal testing, rather than using the technologies in the interest of the animals by enabling positive affective states [50,51,53]. We believe that, for each animal experiment, the benefits of the technology (for monitoring welfare and improving the quality of experimental results) should be carefully weighted with the existing ethical concerns. This weighing should preferably be done by the relevant committee(s) that perform(s) the ethical assessment of the overall experiment.

Third, large quantities of sensitive data are produced, requiring sufficient capacity to store the data in a protected environment, as well as adequate data analysis skills to translate raw data into meaningful information. We recommend educating (future) veterinarians and technicians about the opportunities and challenges of sensor technologies and training them in (basic) data analysis skills, while we also advocate for close collaboration with trained data scientists for more complex cases. Regarding the size of data sets, we, for example, produced video files of 10 Gb a day (recordings with a fps of 25, resulting in 2.16 million frames, each of ~2 million pixels, per day) and accelerometer data of 0.5 Gb per day (three accelerometers measuring three axes at 25 Hz, resulting in 19.44 million data points per day). A straightforward strategy to reduce the data quantity is to lower the sampling frequency. For example, we used accelerometers at 25 Hz, but with a lower frequency we expect we would have reached similar insights. Although the ODBA and VDBA are typically calculated from accelerometer data of 10 Hz or higher, Halsey et al. [54] indicated that this may be severe oversampling and concluded that frequencies as low as 1 Hz may be sufficient, even for smaller animals. The result of Halsey et al. should be further validated in studies that consider the effect of sampling frequency on the long-term monitoring of activity patterns in a variety of species. Note that, when accelerometers are used to detect specific behaviours, rather than general activity, higher sampling frequencies are needed [55]. For our video analysis of the time spent drinking, we down sampled the videos from 25 fps to 2 fps to reduce computation time. In hindsight we could have recorded at 2 fps, which would have been more than sufficient for our purpose considering that most drinker visits lasted more than 5 s. A second strategy to reduce data quantities is to automatically process the data in (semi-) real-time and store only ‘meaningful’ results and discard raw data. For this purpose, regular data transfer and direct data processing are needed. Although the MOX 1 accelerometers that we used in this study are data loggers, various commercially available accelerometers can measure in real-time and transfer the acceleration signals to a nearby device or to the cloud. However, regular data transfer reduces battery life and, consequently, devices would have to be replaced more frequently, causing a higher impact on the animals and increasing the workload for the animal caretakers. Alternatively, a larger and heavier battery can be used, which also causes a higher impact on the animals. As another solution to maintain battery life without increasing the battery size, edge-computing (i.e., performing the computation on pre-programmed devices and only transferring the results instead of raw data) can be applied [56]. Edge-computing is a promising solution, with the downside that one needs to know a priori which results are of interest. 

Fourth, the cost-benefit analysis of sensor technologies should also include the affordability (in terms of financial and time investments) and sustainability (in terms of re-usability and environmental impact). Today, in 2024, video hardware can be rather cheap. For example, a surveillance camera with reasonable specs costs less than 100 euros and a network video recorder costs a few hundred euros. However, data storage and data analysis—depending on the models used—can be computationally intensive, which is unfavourable from a financial and time perspective, as well as from an environmental perspective (e.g., high energy consumption with deep learning models). Moreover, installation and processing steps may be time-consuming. Body-worn sensors may be less expensive in terms of data storage and analysis, but the devices, such as the accelerometers we used, may be a few hundred euros each, although many cheaper devices exist as well, depending amongst other things on the measurement frequencies, battery life, and associated software. If devices can be recharged and re-used in multiple experiments, they are a long-term investment. However, for infection studies under high containment conditions, re-using devices may be undesired or at least require that the devices have high standards in terms of waterproofness (e.g., IP68), such that they can be thoroughly disinfected. For both video cameras and body-worn sensors, there is an environmental impact in terms of e-waste and emissions in the manufacturing process and when disposing of the equipment. 

Fifth, and most importantly, the validity of measurements should always be carefully considered. For any sensor, it is of the utmost importance to understand what it is measuring, and whether this is indeed what we intend to monitor. Activity is a rather broad term and should be interpreted with respect to how it is computed. For example, the ‘distance moved’ can be estimated with video tracking, with UWB coordinates, or with RFID readings at antennae across the pen, resulting in an interpretable measure (e.g., in meters per hour), albeit with varying measurement errors and assumptions across technologies [57,58]. With accelerometers, however, the distance moved is difficult to estimate [59]. Deriving animal activity from an accelerometer relies on the principle that an animal’s acceleration (in one or more of the three axes) fluctuates when moving, even when the animal is moving at a rather constant speed. For example, when walking, the animal experiences accelerations and decelerations throughout the gait cycle. These fluctuations are captured with the VDBA, which uses ‘dynamic accelerations’ and corrects for a static component that is largely determined by gravitational acceleration across the three axes (which, in turn, depends on the orientation of the device). Both ODBA and VDBA have been shown to correlate well with oxygen consumption ($\dot{V}O_{2}$) and speed in a variety of species, with R^2^ values generally above 0.8 [29,60]. It is important to realize that VDBA does not only capture locomotor activity, but also other movements, such as body shaking or turning. Depending on the position of the device, the VDBA might be more or less affected [13]. In this study, we attached the accelerometers to the back of the rams, which is expected to monitor $\dot{V}O_{2}$ well since it is close to the animal’s centre of gravity (as recommended by Wilson et al. [13]). Other attachment positions, such as the ear tag (which the animals already have for identification), may be more user-friendly and less impactful for the animal. However, one should carefully consider that the measured “activity” then also includes other (non-locomotor) movements such as head rotations or ear twitches, and comparison studies are needed to address the impact of the attachment position on monitoring activity patterns through VDBA. 

Regarding drinking behaviour, our approach also has some limitations. We quantified the number of visits and time spent drinking above the drinker, using CV approaches, and obtained individual time series (Figure 6) that corresponded well to the group level results from the water meter (Figure 4D). However, an animal that is moving its head above the drinker is not necessarily drinking, as we also observed in our preliminary analysis, and an animal not moving its head above the drinker while drinking is sometimes not detected (Table S1). Based on our validation video, we expect that we overestimated the time spent at the drinker (Table S1), but that this overestimation was consistent across the experimental period. Another limitation in our approach was that 16% of drinking bouts had multiple rams detected, and we divided the time across rams proportionally to the number of detections. To improve the assignment of bouts to individual rams, one could consider the orientation of the Aruco with respect to the drinker, such that, when one animal’s Aruco is facing the drinker, whereas the other’s is not, all the detected time would be assigned to the former animal. However, in our validation video we noticed that the animals may also drink while standing sideways next to the drinker and drink with a rotated head. Thus, this would require quite a few additional assumptions, probably with rather limited improvement of the results. As an alternative to CV, one could use UWB or RFID sensors and use the time spent in the vicinity of a drinker as a proxy of drinking time [61]. All these approaches may be sufficient to monitor an endpoint that is formulated as “the animal is no longer able to go independently to the drinker”. However, they are insufficient for an endpoint that is defined as “the animal does not drink anymore”, as they only measure proximity to the drinker and do not measure actual water consumption. As a solution, we would advocate for the use of individual drinking bins or use a single drinker with a continuous water meter (that, e.g., automatically transfers data every 10 s) in combination with an RFID, Aruco, or other identification method. 

In summary, sensor technologies have added value for monitoring behaviour in animal infectious disease research, providing continuous, individual, detailed, and objective data that can also be automated. We found various additional insights as compared to routine observations. Despite the limitation of a small group size (*n* = 3), the changes in activity patterns were statistically significant on the group level. Nevertheless, further studies are needed to confirm the observed effects of *T. gondii* infection on sheep behaviour. While we focused on activity and drinking behaviour, similar benefits of using sensor technologies are expected for other behaviours or physiological parameters, such as temperature and respiration. However, even when some of the challenges of sensor technologies are overcome, we expect that there remains a need for careful set-up of these technologies, as well as the interpretation of results by animal caretakers/researchers. Thus, these tools are an addition to routine monitoring approaches, not a replacement. 

## 5. Conclusions

Following infection with *T. gondii*, rams developed fever and showed associated sickness behaviours; among others, a reduced daytime activity (60–80% of the original vectorial dynamic body acceleration) and a reduced drinking behaviour (less than half of the original drinker visits). After recovery from the fever, activity levels increased (with 110–160% of the original vectorial dynamic body acceleration). While we recognize the limited conclusiveness of this case study given the small number of animals, the implemented sensor technologies had various added benefits for behavioural monitoring. By providing continuous, individual, detailed, and objective time series, they offered insights that were not detected with the routine monitoring procedures and allowed for better monitoring of scientific and humane endpoints. Despite their challenges, we recommend wider implementation of such technologies to further improve the quality of experimental results and refine animal experiments. 

## Figures and Tables

**Table 1:** Group-level comparison of behaviour before and after infection. For each day after infection, the ram’s mean behaviour was compared to the ram’s mean behaviour for all days before infection. The average difference across rams was calculated in trait values (Delta), and as a percentage of a ram’s mean behaviour before infection (Delta %). *p*-values were obtained for two-sided paired *t*-tests (*p*-values below 0.05 are shown in bold). The traits were the vectorial dynamic body acceleration (VDBA), the time spent at the drinker (in seconds per hour), and the number of drinking bouts.

	VDBA			Time at Drinker (s/h)			No. Drinking Bouts		
Day	Delta	Delta %	*p*-Value	Delta	Delta %	*p*-Value	Delta	Delta %	*p*-Value
0	−0.0011	−2	0.259	−2.5	−13	0.177	−0.181	−17	0.047
1	−0.0016	−3	0.297	0.9	4	0.247	−0.028	−2	0.315
2	NA *	NA *	NA *	3.0	14	0.208	0.181	16	0.233
3	−0.0003	0	0.349	NA *	NA *	NA *	NA *	−45	NA *
4	−0.0031	−7	0.084	−7.1	−38	**0.021**	−0.319	−33	**0.014**
5	−0.0078	−19	**0.002**	−8.8	−48	**0.020**	−0.403	−40	**0.003**
6	−0.0084	−21	**0.007**	−8.9	−49	**0.015**	−0.514	−51	**0.015**
7	−0.0069	−17	**0.009**	−6.9	−37	**0.002**	−0.264	−25	0.109
8	−0.0081	−20	**0.003**	−6.7	−36	0.063	−0.347	−35	**0.017**
9	NA *	NA *	NA *	−4.5	−25	**0.019**	−0.194	−17	0.246
10	−0.0021	−5	0.093	−3.0	−18	0.226	−0.278	−29	0.080
11	0.0018	4	0.081	−0.9	−5	0.346	0.056	3	0.339
12	0.0025	6	**0.022**	7.6	41	0.123	0.375	34	0.146
13	0.0036	9	**0.021**	5.6	30	0.177	0.139	12	0.266
14	0.0059	15	**<0.001**	8.9	45	0.083	0.431	43	0.050
15	0.0054	14	**<0.001**	NA *	NA *	NA *	NA *	NA *	NA *
16	0.0034	9	**0.028**	NA *	NA *	NA *	NA *	NA *	NA *
17	0.0070	18	**<0.001**	NA *	NA *	NA *	NA *	NA *	NA *
18	0.0072	18	**0.003**	11.3	55	0.167	0.708	67	0.116
19	0.0075	19	**0.013**	13.1	67	0.087	0.403	41	**0.018**
20	0.0072	18	**0.014**	9.4	50	**0.006**	0.625	64	**0.007**
21	0.0055	14	**0.002**	18.2	98	**0.002**	0.694	71	**0.001**
22	0.0006	2	0.256	−3.9	−22	**0.030**	−0.148	−14	0.327

* Not applicable, due to missing data on this day.

## Data Availability

The datasets generated and analysed during the current study are not publicly available due to sensitivity of the data and privacy of the animal caretakers, but (particularly accelerometer) data are available from the corresponding author on reasonable request.

## Supplementary Materials

The following supporting information can be downloaded at: https://www.mdpi.com/article/10.3390/ani14131908/s1, Table S1: Comparison of 34 manually observed drinking bouts with automatically detected bouts for a single 24-h video; Figure S1: Rolling average (of 8 h) of the vectorial dynamic body acceleration (VDBA) for each ram over time, expressed as a percentage (%) of the mean VDBA for the corresponding hour in the days prior to inoculation; Figure S2: Rolling average (of 8 h) of time spent at the drinker and number of bouts for each ram over time, expressed as a residual compared to the mean for the corresponding hour in the days prior to inoculation.
