# Supplementary figures and images for: Added Value of Sensor-Based Behavioural Monitoring in an Infectious Disease Study with Sheep Infected with Toxoplasma gondii
Source: Animals (Basel). 2024 Jun 27;14(13):1908. doi: 10.3390/ani14131908 (PMC11240357; doi:10.3390/ani14131908)

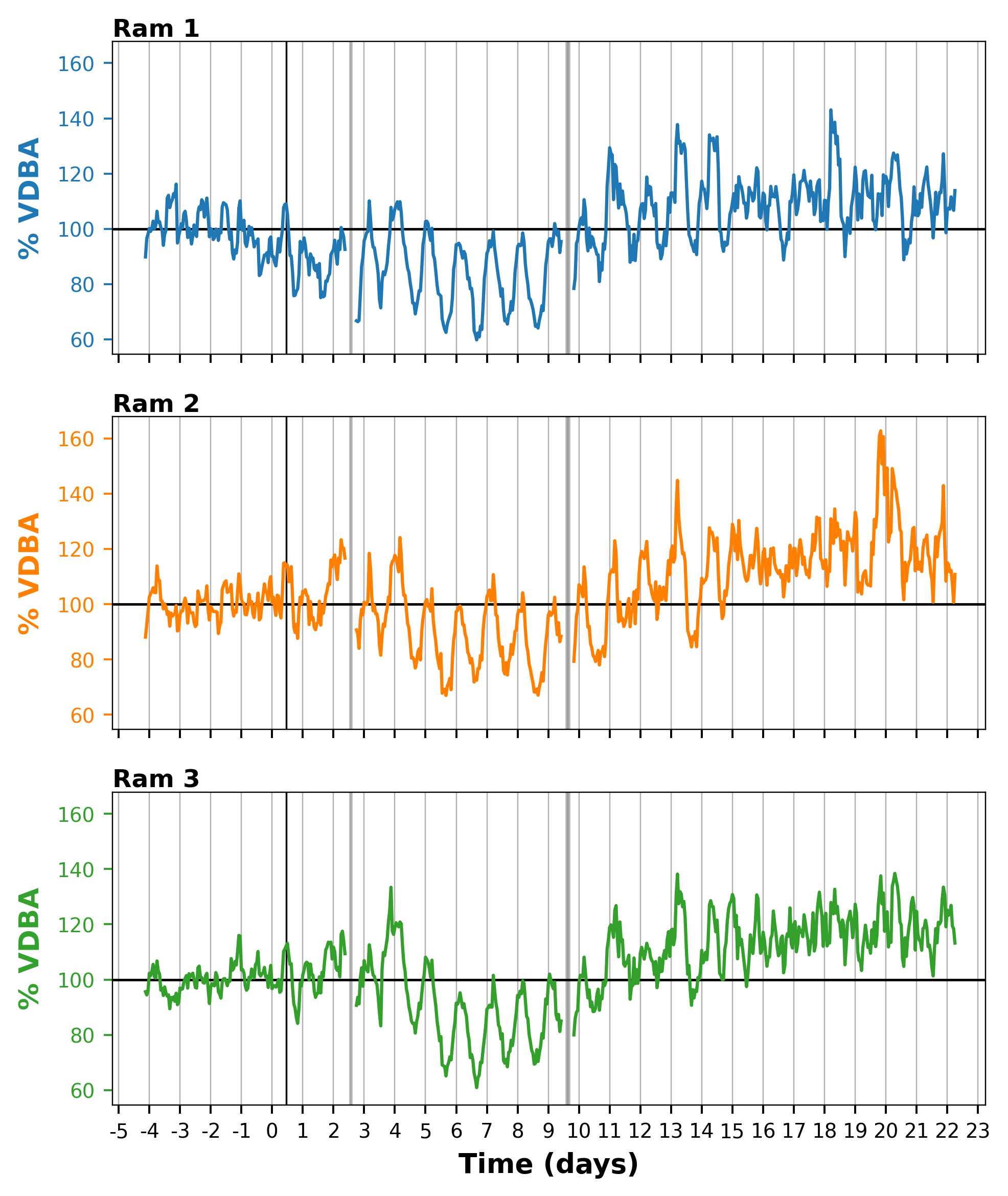

Supplement: Supplementary file 1 [file animals-14-01908-s001.zip › Figure_S1.png]

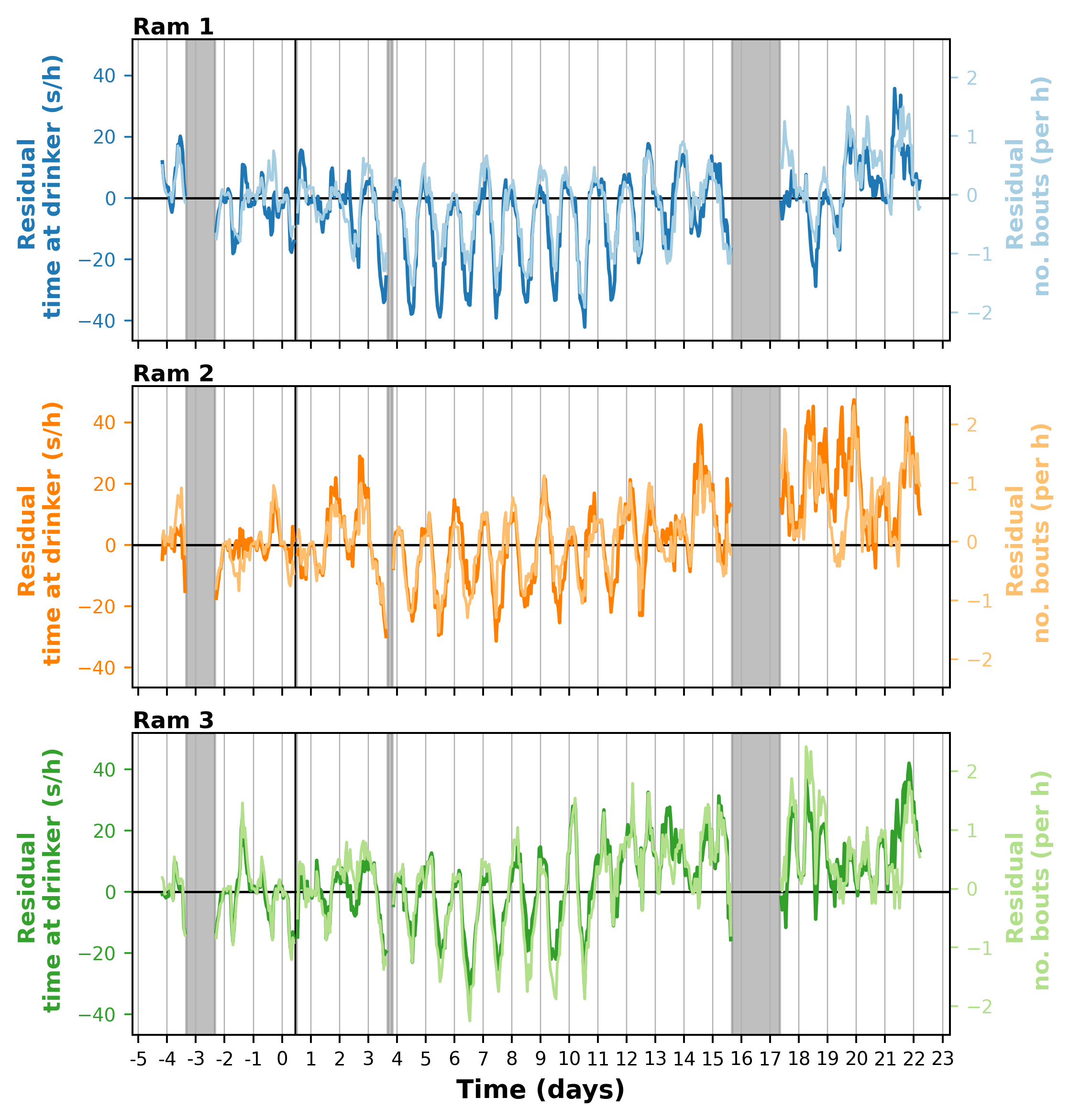

Supplement: Supplementary file 1 [file animals-14-01908-s001.zip › Figure_S2.png]
